# Validation of wing geometric morphometrics in *Chrysodeixis* spp. (Lepidoptera: Noctuidae) to support pest identification in invasive species survey programs

**DOI:** 10.3389/finsc.2025.1542467

**Published:** 2025-01-28

**Authors:** Allan H. Smith-Pardo, Karina M. Torres, Silvana V. Paula-Moraes

**Affiliations:** ^1^ Plant Identification Technology Laboratory (PITL), Science and Technology (S&T), Plant Protection and Quarantine (PPQ), Animal and Plant Health Inspection Service (APHIS), United States Department of Agriculture (USDA), Sacramento, CA, United States; ^2^ Entomology & Nematology Department, West Florida Research and Education Center, University of Florida, Jay, FL, United States

**Keywords:** domestic surveys, pheromone trapping, tomato looper, soybean looper, wing landmarks

## Abstract

Looper moths of the genus *Chrysodeixis* (Lepidoptera: Noctuidae: Plusiinae) are important pests of many crops and native plants worldwide. *Chrysodeixis chalcites* (Esper) is listed as an invasive species for the United States with records of interception. Native species of the Plusiinae subfamily are morphologically similar and commonly cross-attracted in survey trapping programs for *C. chalcites*, such as *Chrysodeixis includens* (Walker), a native economic pest. The species identification relies on male genitalia dissection and DNA analysis. These processes are time and cost-consuming and require expertise. In this work, we evaluated the use of wing geometric morphometrics (GM) as a tool to overcome the identification challenges associated with the complex morphologies of *Chrysodeixis* spp. The cleaned wings of specimens with validated identification were photographed under a digital microscope, and seven venation landmarks were annotated from the images. The digital coordinates of the wing landmarks were analyzed in MorphoJ. Our results validated the use of GM for distinguishing the invasive *C. chalcites* from the native *C. includens*. A limited number of landmarks on the center of the wing was used to address the challenges in GM for trap-collected lepidopteran pests. Future automation of the novel application of GM for identifying *C. includens* can be explored in trapping systems for IPM and surveys for the invasive *C. chalcites*.

## 1 Introduction

Moths of the genus *Chrysodeixis* (Lepidoptera: Noctuidae) belong to the Plusiinae subfamily. The number of species in the genus *Chrysodeixis* depends on the authority, but a recent search on the Animal Diversity Web listed 46 species (1). This genus is primarily Paleotropical, with most species inhabiting northern Africa, the Mediterranean region, and the Middle East. There are some species in southeast Asia as well as Oceania, and at least one species native to the Western Hemisphere (2). The genus was first described by Hübner in 1821, and limited taxonomic reviews have been conducted since (3). In a review of all known plusiines in the world, Kitching (2) reclassified the genus *Pseudoplusia* (McDunnough) as a subgenus of *Chrysodeixis*, establishing the *Pseudoplusia* subgenus of *C. includens* (Walker) and *C. dalei* (Wollaston). Recently, *C. dalei* was found to be synonymous to *C. includens* (4). Kostrowicki (5) proposed to separate the previously conspecific complex of *C. chalcites* (Esper) and *C. erisoma* (Doubleday). While there was an initial disagreement over the classification, later studies supported the distinction using DNA barcoding analyses (6, 7). The complication of species identification and taxonomy of *Chrysodeixis* is attributable to the high degree of similarity in external morphology.

The adults of all moth species of the Plusiinae subfamily are similar in general appearance, with a few exceptions (8). Adult plusiines can be recognized by the presence of strong thoracic and abdominal tufts of setae and by characteristic gold or silver markings on the forewing. Such markings can be variable and are unreliable for species-level identification purposes. According to Twinkle et al. (9) and Kostrowicki (5), *C. chalcites* is easily identifiable and distinguishable from *C. erisoma.* Other sources indicate *C. chalcites* and *C. erisoma* are externally identical (10) and extremely difficult to identify using morphologic features (11). Similarly, the wing pattern morphology of *C. includens* appears similar to that of *C. chalcites*, and both species are described to exhibit intraspecific wing pattern variations (6, 12). Male genitalia dissections and DNA analyses are the most reliable methods of identifying *Chrysodeixis* spp. (10, 13).

*Chrysodeixis chalcites* is recognized as a serious lepidopteran pest in many regions around the world, including Europe, the Mediterranean, the Middle East, and Africa (14). This species is listed as an economic pest in Colombia, Costa Rica, Japan, Ecuador, Nicaragua, Peru, the Eurasian Customs Union, and the Republic of Korea (15). The larvae are extremely polyphagous, feeding on foliage and reproductive parts of the host plants growing outdoors and in protection, including both shade and greenhouses. This species can cause severe damage in many cultivated plant families, including Acanthaceae, Asteraceae, Bignoniaceae, Boraginaceae, Brassicaceae, Convolvulaceae, Crassulaceae, Lamiaceae, Fabaceae, Malvaceae, Orchidaceae, Rosaceae, Scrophulariaceae, Solanaceae, Verbenaceae and Violaceae. The major reported hosts are tomato, soybean, short-staple cotton, tobacco, beans, and potato (14). In addition, the following crops are also listed as host plants of *C. chalcites*: alfalfa, artichokes, cauliflower, cabbage, chrysanthemum, *Citrus* spp., clover, corn, sweet pepper and other greenhouse and field fruits and vegetables, ornamental plants, and wheat (16). From 1984 to 2014, specimens of *C. chalcites* have been intercepted at U.S. ports over 300 times from various plants and countries of origin (16). *Chysodeixis chalcites* is listed as an invasive pest in the United States, and there are reports of establishment in the counties of southwestern Ontario, Canada (17). On February 22, 2019, a USDA Plant Pest Identification Notification was issued upon a confirmation of two samples positive for *C. chalcites* in samples from Michigan. Based on the high risk of introduction in the U.S. (16), this species is currently listed as having quarantine importance and continues to be targeted in state surveys using sex pheromone trapping by APHIS. However, the sex pheromone commercial formulations used for the detection of *C. chalcites* in bucket traps are not specific and yield a high level of cross-attraction of four morphologically similar native plusiines, including *C. includens* (12).

*Chrysodeixis includens* is a native economic pest in the U.S. that feeds on over 174 species of host plants across 39 families (18). This species occurs in the U.S. as far north as Maine and south to Texas, but it is most abundant in the southeastern regions of the country (19). *Chrysodeixis chalcites* and *C. includens* are allopatric species and similar in appearance and wing patterns (11). Because the external morphology of both species is identical, they can only be segregated by DNA barcoding or dissection of the male genitalia (10). The process of dissecting male genitalia is time-consuming and requires expertise not only to dissect and clean the structures but also for the correct identification of the species. This complexity also limits the number of specimens that can be accurately identified in a given time. Furthermore, this method does not apply to the identification of females; while uncommon in pheromone traps, they may be collected by other means. Similarly, DNA analysis is a demanding process that is unideal for timely pest survey and mitigation decisions.

Studies using geometric morphometrics (GM) analysis have revolutionized the way researchers perform taxonomy studies for species identification (20–23). Based on mathematical representation of shape and associated statistical analysis of the shape variation using Procrustes analysis, GM has provided accuracy, particularly when dealing with closely related species (24, 25). The objective of this study was to validate morphometric shapes of the invasive *C. chalcites* and the native *C. includens* using seven landmarks around the center of the right forewing. Morphometric analyses were also performed in three other native plusiines that are commonly cross-attracted in pheromone trapping for *C. chalcites*: *Trichoplusia ni* (Hübner), *Rachiplusia ou* (Guenée), and *Ctenoplusia oxygramma* (Geyer). Overall, the goal of the study was to improve the identification resources in APHIS pest survey programs for *C. chalcites* by streamlining the screening process of large numbers of cross-attracted native plusiines.

## 2 Materials and methods

### 2.1 Insect collection

The specimens used in the study included images of *C. chalcites* and *C. includens* provided by USDA-APHIS-PPQ and specimens of *C. includens* collected at West Florida Research and Education Center (WFREC), the University of Florida, Jay, FL, and from commercial fields in the Florida Panhandle, a region with a high diversity of plusiines (12). Collection methods included bucket and Trécé delta (Trécé Inc Pherocon VI trap, Adair, OK) trapping baited with sex pheromone lures, and field collection of larvae and pupae. The bucket and delta traps were located at least 100 m apart along the edges of commercial peanut and soybean fields. Only specimens with well-preserved right forewings were collected from the traps for analysis. From Delta traps baited with *C. includens* lures at the WFREC and commercial fields, 22 specimens were recovered between 2017 and 2021, and 121 specimens were recovered from bucket traps in 2019. Additionally, 72 plusiines were collected year-round from the WFREC and commercial fields from bucket traps baited with *Autographa gamma* and *C. chalcites* lures in 2023 and 2024. Finally, drop cloth sampling in soybean fields at the WFREC was conducted to further increase the number of *C. includens* for the study. All observed larvae and pupae were collected during the sampling effort in August 2023. Each specimen was placed in individual cups and reared on a multispecies lepidopteran diet (Southland Products, Lake Village, AR) in the Entomology Lab at the WFREC. A total of 96 specimens were successfully reared and stored in a 1°C refrigerator.

### 2.2 Species validation

The species identification of the 23 specimens of *C. chalcites* and 24 specimens of *C. includens* provided by APHIS were performed based on male genitalia dissection. The 311 plusiines collected at the WFREC and commercial fields were identified via real-time PCR testing for *C. includens* following the assay described in Zink et al. (7). Six specimens did not yield identification results or were not plusiines, so they were not used for the analysis. 242 specimens were confirmed to be *C. includens*. The remaining 63 specimens were identified as one of three native plusiine species: *R. ou*, *T. ni*, or *Ct. oxygramma*. Few specimens of *Ct. oxygramma* were collected (n = 3) because *Ct. oxygramma* is easily distinguishable from other plusiines (12) and thus removed from our trap collection. *Trichoplusia ni* was caught in low abundance (n = 2) because the trapping dates did not occur during *T. ni* peak flying phenology (12). Regarding the sex of the moths used in the study, all 23 specimens of *C. chalcites* were male. In the case of *C. includens*, there was a total of 194 male specimens, 56 female specimens, and 16 specimens that were unable to be sexed due to damage on the abdomen.

### 2.3 Wing processing

The right forewings of the 47 specimens provided by APHIS (*C. chalcites* and *C. includens*) were cleaned using a paintbrush with a solution of 50% hypochlorite and rinsed with water. After the wings were cleaned of scales, they were laid on a microscope slide to dry out. Once dry, the wing was covered with another slide, tightened using tape on two opposite sides, and labeled with the species, locality, and date. For the 242 specimens from WFREC and commercial fields, the right forewings were removed from each specimen, and the sex was recorded. A modified method was used for the processing of specimens. On a petri dish, each wing was dipped in 70% isopropyl alcohol solution and then transferred to a separate solution of 50% hypochlorite. The wings were held in the solution for no longer than two minutes to avoid damage. A fine-tipped paintbrush was used to thoroughly rinse the wings in water and remove the scales and chemical solutions. Once cleaned, wings were dried using low-lint cellulose wipers and returned to their respective 2mL centrifuge test tube. Because forewings collected from Delta traps and kept on sticky liners often sustain a glue residue on the wing, these glue residues can compromise the efficient removal of scales using hypochlorite and alcohol. The residue was removed from the wings using a household glue solvent (Goo Gone Original, Gurnee, IL), and it was a particularly important step in the study of landmarks for GM analysis. After the wings were cleaned following the procedures described above, a few drops of the glue solvent were placed on the wing, and the residue was removed with a paintbrush. The wings were rinsed in water to ensure all the solvents and scales were cleaned from the wing.

### 2.4 Wing imaging

**Figure 1:**
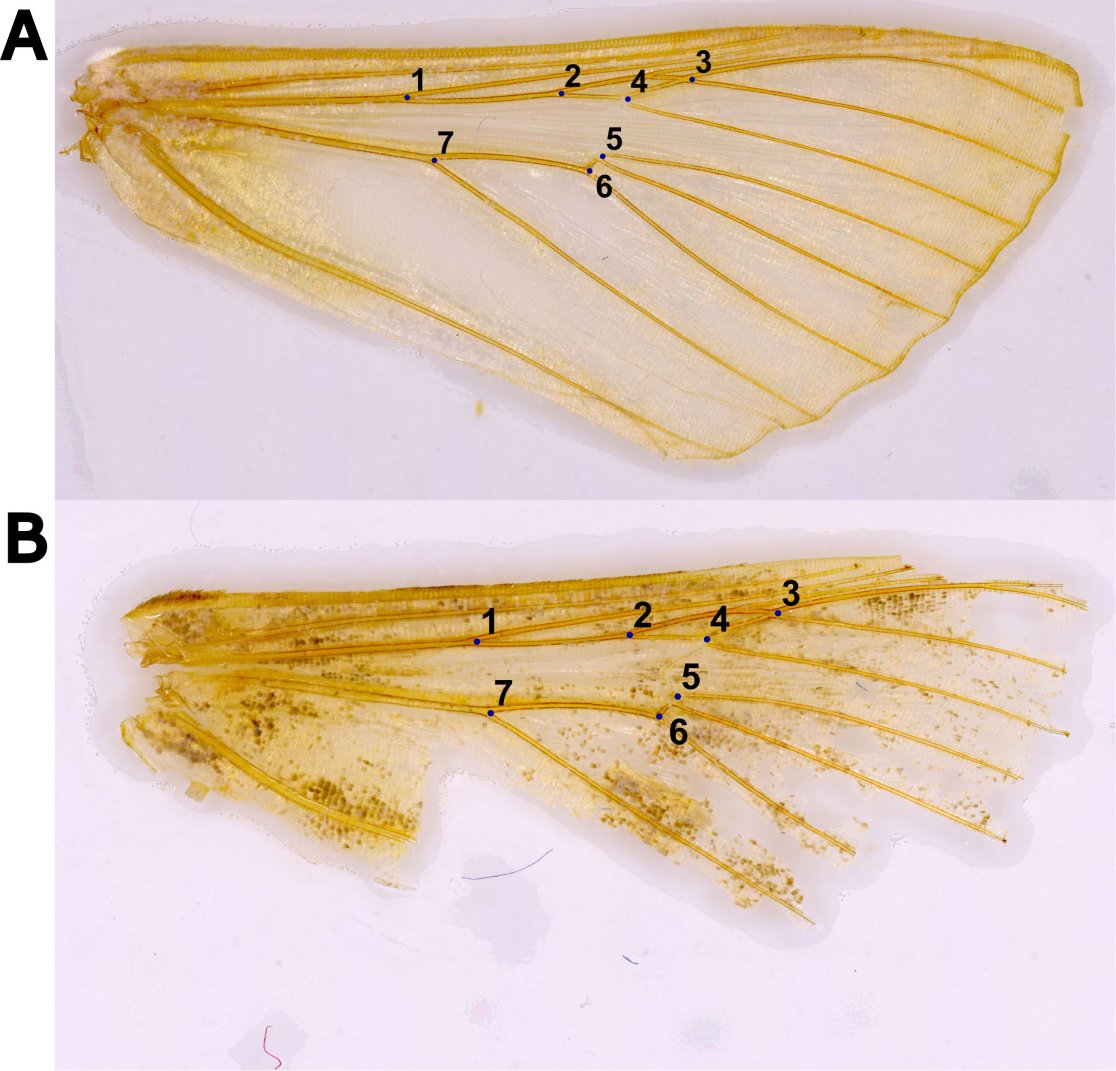
Two cleaned right forewings with the seven landmarks placed around the discal cell and radial veins, including **(A)** an ideal male specimen of *Chrysodeixis includens* reared in the lab and **(B)** a specimen of *Trichoplusia ni* collected from a Delta trap with damage along the wing margins.

For the wings collected from APHIS surveys, slides were photographed in the laboratory using a Nikon SMZ18 dissecting scope with a DS-Fi3 camera at a magnification of 5-10x at the Pest Identification Technology Laboratory, USDA-APHIS-PPQ, Sacramento, California. The images of the cleaned wings were processed using Adobe Photoshop CS with edits including autotune, auto contrast, and sharpening to obtain a clear rendering of the veins. Adjusted images were saved in grayscale. Based on this work of initial imaging with the above specimens, it was indicated that higher-quality images would be more beneficial for larger-scale and precise applications of the study. As a result, the majority of the cleaned right forewings were imaged individually using an imaging system (ZEISS Smartzoom 5) at a magnification of 140x in the Entomology Laboratory at WFREC/UF. Each wing was secured and flattened on a microscope slide with a clean piece of packing tape. The image system was used to standardize and replicate images. An optimal image condition was set for all samples, including color saturation, noise filtering, sharpening, brightness, and contrast adjustments, as well as a Realtime High Dynamic Range (HDR) filter. As a result, high-resolution images were produced (**Figure 1**).

### 2.5 Wing landmarking

A total of seven landmarks were established around the Discal cell and along the intersections of the Radial (R), Radial Sectorial (Rs), Medial (M), and Cubital (Cu) veins, (**Figure 1**). The center of the wing was chosen for the analysis because the wing margins are more vulnerable to damage (**Figure 1B**). For moths collected in traps, the center of the wing is usually preserved and thus more reliable for analyses. Images of the wings were compiled into sets of 20 images to maximize time and computer processing efficiency. The program tpsUtil on version 1.83 (26) was used to assemble images. Landmark coordinates were then manually annotated using tpsDig2 on version 2.32 (27). Because the data format used by tpsDig2 and the analysis software are dissimilar, the landmark data must be formatted before importing to MorphoJ (28). A Python script was written to automate the formatting of the data (29). All morphometric analyses were performed using the program MorphoJ.

### 2.6 Data analysis

Once the landmark coordinates were obtained for all specimens, three datasets were created for separate analyses. One dataset consisted of only *C. chalcites* and *C. includens* specimens, and another consisted of all collected plusiine specimens (*C. chalcites*, *C. includens*, *R. ou*, *T. ni*, and *Ct. oxygramma*). These datasets were referred to as the *Chrysodeixis* dataset and the plusiine dataset, respectively. The third dataset, or the *C. includens* dataset, was a subset of the *Chrysodeixis* dataset that was used to test for sexual dimorphism in *C. includens*. Specimens that were unable to be sexed were not included in the analysis of the *C. includens* dataset. Geometric morphometric analyses necessitate at least twice the number of individuals than landmarks (30). Because the number of specimens for *T. ni* and *Ct. oxygramma* did not meet these requirements, these two species were included in the analysis to demonstrate the possible clustering of their wing shape relative to other plusiines, but their significance is not reported in the results.

For the *Chrysodeixis* dataset, the shape information was extracted with a Procrustes fit analysis (31, 32), aligned by the principal axes. To identify if there was any size effect on the shape data between the two analyzed species, multivariate regression was performed using the shape coordinates as a dependent variable and centroid size as an independent variable, and a permutation test using 10,000 randomization rounds was performed to identify if there any statistical size effect. The shape variation was analyzed in the entire dataset using a principal component analysis (PCA) based on the covariance matrix of the individual wing shape. The differences between groups were determined with a Procrustes ANOVA, using the species as a classifier. Finally, a canonical variate analysis (CVA) was run using 10,000 permutation tests and the species as a classifier to identify the shape features that best differentiated each species.

The plusiine dataset was examined using a Procrustes fit, multivariate regression, PCA, ANOVA, and CVA as described above. An additional ANOVA and CVA were performed using the genus as a classifier. The *C. includens* dataset was also analyzed with a Procrustes fit, PCA, ANOVA, and CVA using the sex of the specimen as a classifier.

## 3 Results

### 3.1 *Chrysodeixis* dataset

The first examination of the shape data was performed using the evaluation of allometry for *C. chalcites* and *C. includens*. The result of the multivariate regression indicated that there is a significant influence (*P* < 0.001) of allometry on the data, where 15.8999% of the data is predicted to be influenced. However, the allometry was distinct between imaged specimens provided by APHIS and specimens imaged at the WFREC. The differences in image quality and size between imaging methods produced misleading results by the multivariate regression. When the regression was performed using only the APHIS specimens (23 C*. chalcites* and 24 C*. includens)*, the allometry between species was not significant (% predicted: 0.7244, *P* = 0.8399).

**Figure 2:**
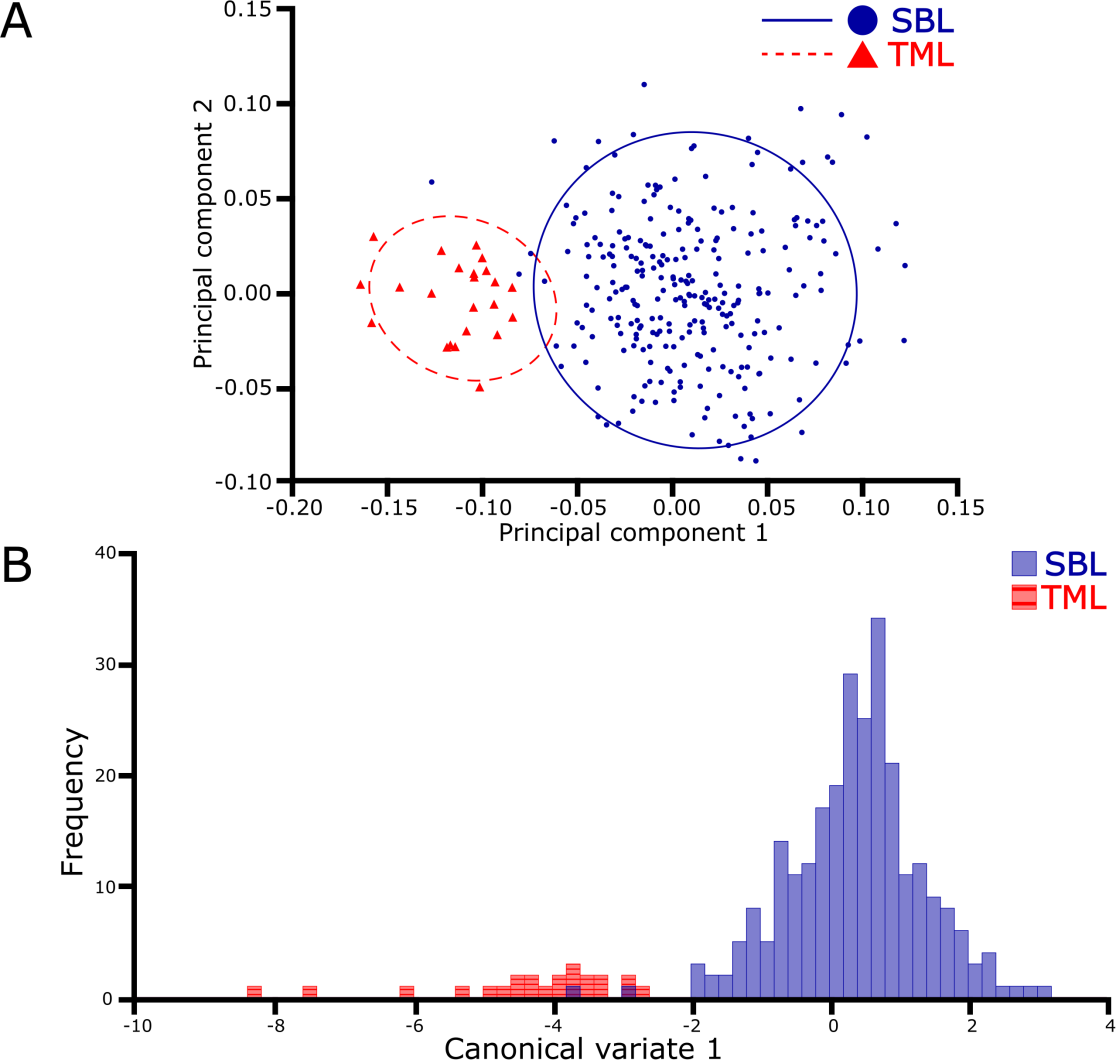
Visualizations of the principal component and canonical variate analyses of the *Chrysodeixis* dataset, including *C. chalcites* (TML) and *C. includens* (SBL), show the distinct clusters of the two species. The first two principal groups **(A)** show the wing shape variation is differentiated between species, and a 90% confidence threshold highlights the respective clusters. Based on wireframe shape, the single canonical variate **(B)** indicates an almost complete separation of each species.

**Figure 3:**
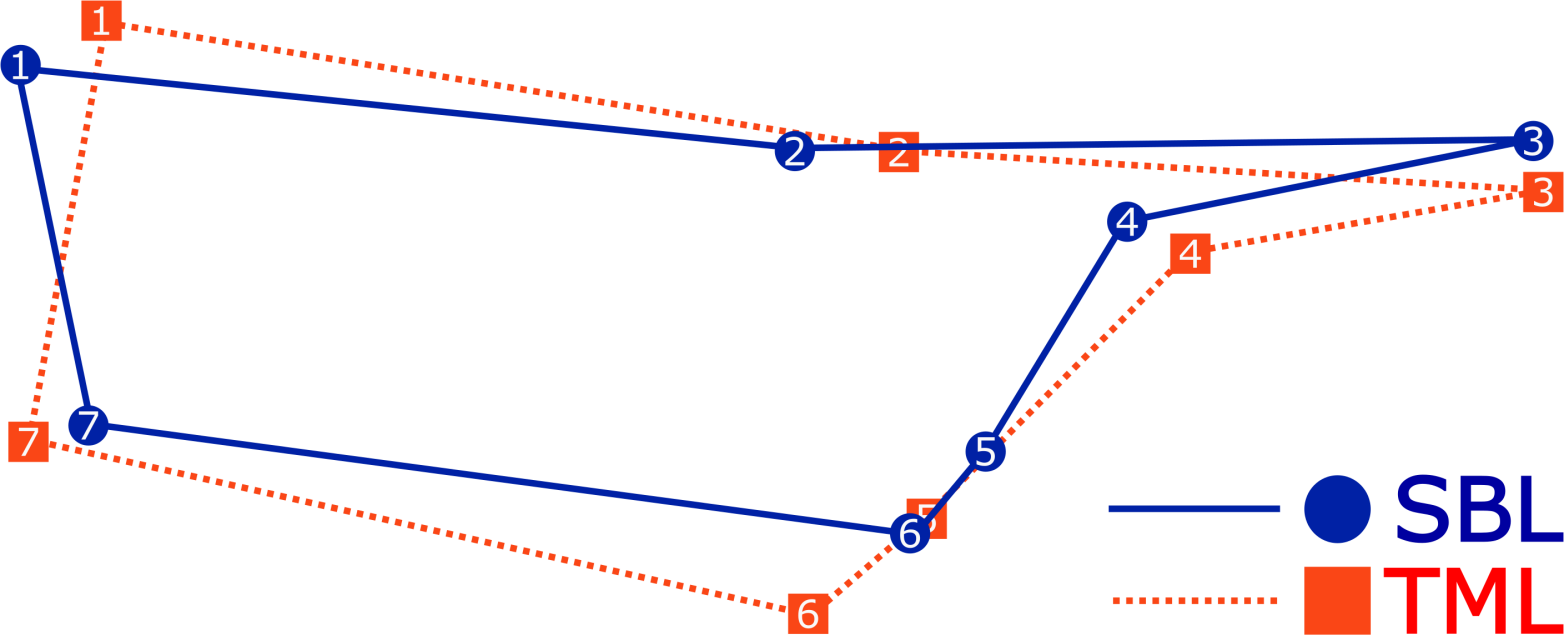
The mean wireframe shape using Procrustes landmark coordinates of *Chrysodeixis chalcites* (TML) and *C. includens* (SBL) demonstrating differences in wireframe shape.

The PCA using the covariance matrix of the individual wing shape found that the first three dimensions of the 10-dimensional shape space accumulated 80.4% of the total shape variation. This variation was primarily attributed to the first principal component (PC1) at 41.8%, followed by PC2 at 22.9% and PC3 at 15.7%. The remaining dimension of the shape space (7) covered 19.6% of the shape variation. The scatterplot of the PCA visualized two principal groups of species where the wing shape variation was differentiated (**Figure 2A**). The principal differences between species were attributed to a change in shape width, where *C. chalcites* revealed a wider wireframe shape than *C. includens*. A comparison of the mean Procrustes coordinates best visualizes the differences in wing shape morphology (**Figure 3**). The landmarks 5, 6, and 7 appeared to widen the discal cell in *C. chalcites*. The landmarks 1 and 7 shared a closer proximity near the basal portion of the discal cell in *C. includens* than in *C. chalcites*. The distance between landmarks 4 and 5 was also reduced in *C. includens*.

The Procrustes ANOVA between the shape coordinates and the species was performed, and the results indicated significant statistical differences for the centroid size (F: 197.77, *P* < 0.0001) and wing shape (F: 67.94, *P* < 0.0001). The CVA demonstrated significant differences between species, both visually (**Figure 2B**) and statistically (F: 67.9411, *P* < 0.0001). The CVA resulted in one canonical variate (CV) accounting for all the variation between species. Two specimens of *C. includens* overlapped in the otherwise distinct cluster of *C. chalcites*.

### 3.2 Plusiine dataset

**Figure 4:**
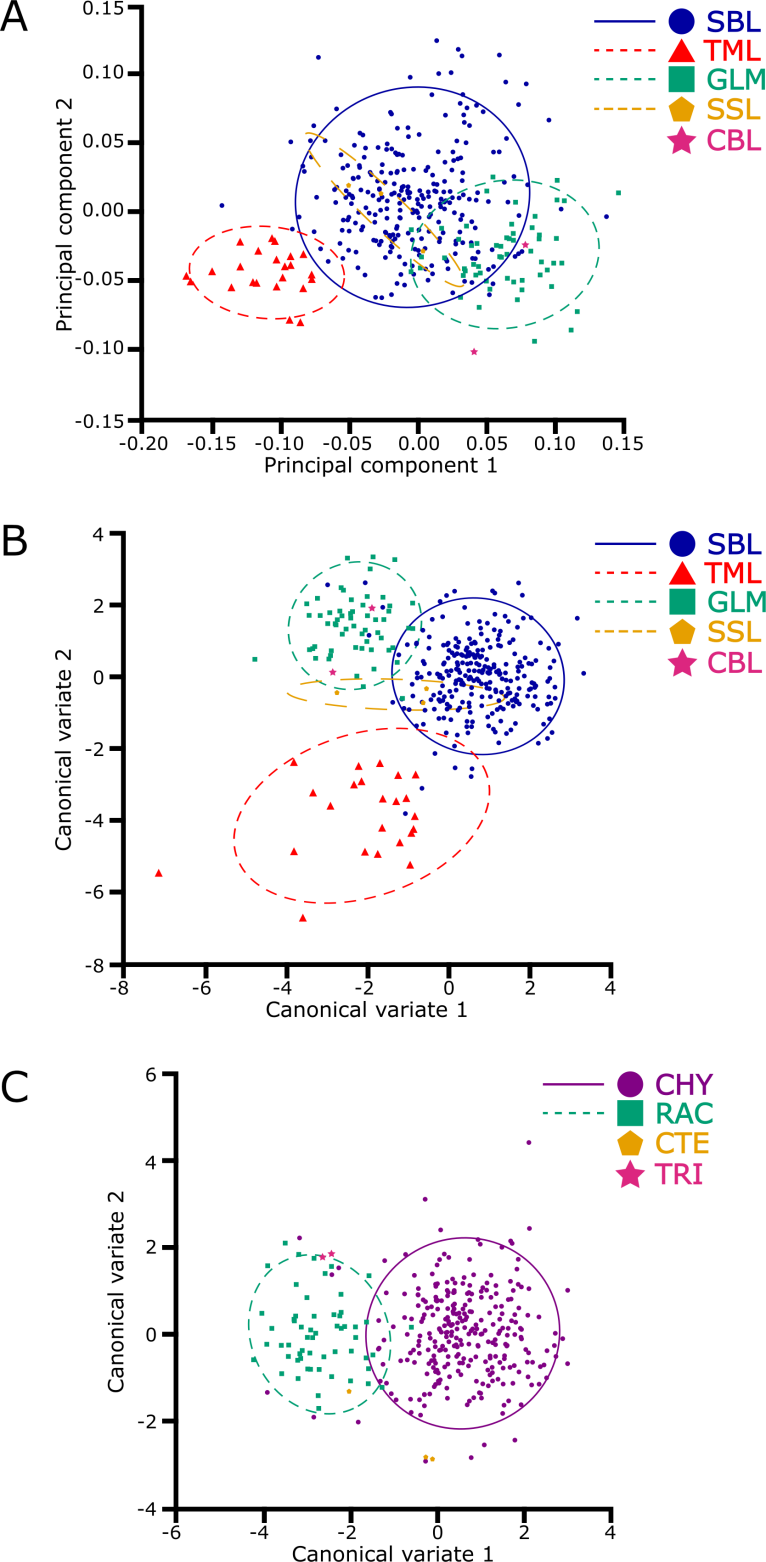
The plusiine dataset demonstrates overlapping clusters of native plusiines and a greater separation in the invasive *Chrysodeixis chalcites* (TML) in the principal component and canonical variate analyses. The first two principal groups **(A)** indicate similarities in shape variation between native plusiines: *C. includens* (SBL), *Rachiplusia ou* (GLM), *Ctenoplusia oxygramma* (SSL), and *Trichoplusia ni* (CBL). When considering species as a classifier, the first two canonical variates **(B)** show greater similarities in shape features of native plusiines than the invasive *C. chalcites*. When considering genus as a classifier, the first two canonical variates **(C)** show separation between the genera *Chrysodeixis* (CHY) and *Rachiplusia* (RAC); the genera *Ctenoplusia* (CTE) and *Trichoplusia* (TRI) are also visualized to show their potential clusters. In each graph, a 90% confidence threshold highlights the respective cluster of species or genera.

The multivariate regression also found a significant influence of allometry on the plusiine dataset (% predicted: 15.8999%, *P* < 0.0001). However, this result is caused by the differences in image quality between APHIS and WFREC specimens. The PCA of the plusiine dataset was similar to the results of the *Chrysodeixis* dataset, where the shape space was distributed to 10 dimensions with 81.0% of the variance captured by the first three principal components: PC1 at 42.6%, PC2 at 23.3%, and PC3 at 15.1%. The visualization of the PCA reveals a distinct cluster of *C. chalcites*, but the cluster of *C. includens* appears to have considerable overlap with *R. ou* (**Figure 4A**). The Procrustes ANOVA of the shape coordinates for each classifier found significant differences in the centroid size and wing shape (**Table 1**). The two CVAs showed considerable differences in the variance captured by each CV, indicating the influence of *C. chalcites* relative to other plusiines. For the CVA considering genus as a classifier, there was a significant difference between genera (F: 24.1436, *P* < 0.0001) with CV1 accounting for 94.6% of the variation and three other CVs making up the remaining 5.4%. For the CVA with species as a classifier, there was a significant difference between species (F: 40.0369, *P* < 0.0001), with CV1 and CV2 accounting for 53.6% and 43.7% of the variation, respectively, and two other CVs making up the remaining 2.7%. Similar to the PCA, the cluster of *C. chalcites* is clearly distinct from other species in the CVA scatterplot (**Figure 4B**). The Procrustes distance for the CVA species comparisons of *C. chalcites* and *R. ou* are nearly twice that of *C. includens* and *R. ou* (**Table 2**). When considering *Chrysodeixis* as a genus in the CVA, there is only a slight overlap between the clusters of *Chrysodeixis* and *Rachiplusia* (**Figure 4C**). The Procrustes distance between the genera *Chrysodeixis* and *Rachiplusia* is similar to the distance between *C. includens* and *R. ou* in the species CVA, likely due to the higher sample size and thus greater influence of *C. includens* relative to *C. chalcites* (**Table 2**).

**Table 1:** Procrustes ANOVA significance results of the plusiine dataset for each classifier (genus and species).

Classifier	ANOVA Centroid Size		ANOVA Procrustes Shape	
	F	*P*	F	*P*
Genus	24.58	<0.0001	24.14	<0.0001
Species	89.27	<0.0001	40.04	<0.0001

**Table 2:** Procrustes distance among groups of *Chrysodeixis* spp., *Rachiplusia* sp., and plusiine genera.

Comparisons	Procrustes distance
*C. includens* vs *C. chalcites*	0.1273
*C. includens* vs *R. ou*	0.0856
*C. chalcites* vs *R. ou*	0.1758
*Chrysodeixis* vs *Rachiplusia*	0.0895

Note that comparisons of species or genera were not included if their sample size was less than fourteen.

### 3.3 *C. includens* dataset

The PCA for the *C. includens* dataset found a 10-dimensional shape space with PC1 at 33.1%, PC2 at 28.5%, PC3 at 19.3%, and the remaining principal components accounting for 19.1% of the shape variation. The Procrustes ANOVA to test for sexual dimorphism within *C. includens* found significant differences in centroid size (F: 7.87, *P* < 0.0054) but not for wing shape (F: 1.58, *P* < 0.1055). The CVA captured all the variation in one canonical variate and found no significant differences between the sex of *C. includens* (F: 1.5825, *P* < 0.1590).

## 4 Discussion

The present study contributes to identification resources for pest species in the subfamily Plusiinae. Precisely, it builds validated GM information to allow differentiation between the invasive *C. chalcites* and the native *C. includens* in survey programs. Wing morphology in insects has been widely used as an important model in ecological, systematic, and evolutionary studies (23, 33–37). This is partly because well-defined landmarks can be established on the wing vein intersections, thus making them very suitable for morphometric analyses (38). The veins are linear structures that are distributed in specific patterns on the wing blade, providing structural rigidity to the wing. These specific characteristics of the insects are suitable traits for GM to discriminate principally cryptic species that are difficult to identify using traditional morphometrics (24, 25). The results of the work evaluate the efficacy of GM using the shape of the forewing venation as a solution to the challenges faced for the identification of species in the *Chrysodeixis* genus.

The Procrustes ANOVA showed a statistical level of significance when comparing the shapes (wing vein intersection wireframe) of *Chrysodeixis* spp. The CV scores clearly showed the difference between the two species and confirmed that it is possible to separate them using the seven landmarks selected in the forewings. Although the forewing shapes of two *C. includens* specimens overlapped in the cluster of *C. chalcites* (**Figure 2**), the majority of the specimens are clearly separated in both analyses. Overall, the results indicated that it is possible to use GM of the forewing venation to distinguish the invasive *C. chalcites* and the native *C. includens*. The GM analyses are also capable of filtering out native plusiines that are cross-attracted in *C. chalcites* trapping programs. In both the PCA and CVA (**Figure 4**), the cluster of *R. ou* wing shape was clearly separated from the *C. chalcites* cluster. Although the clusters of *R. ou* and perhaps other native plusiines overlap the *C. includens* cluster to some extent, the distinct cluster of *C. chalcites* from native plusiines is indicative of the applicability of GM to identify *C. chalcites*, which is critical for survey programs that require a high volume of identification of native plusiines.

The method presented here represents a contribution to survey programs for *C. chalcites.* The use of the validated morphometric shapes in this study does not require expertise in insect anatomy and dissection, and the analysis is less costly than identification methods, such as DNA and genitalia dissection. For the GM process detailed in this study, the preparation of each wing (from dissection to mounting on the slide and image capture) took no more than a few minutes once the procedure was standardized. The landmarking, formatting of coordinates, and analysis took only a few minutes because the number of landmarks was unequivocal (cross-veins) and reduced (only seven per wing). While the process of preparing wings for GM analysis is still laborious, the preparation process can be streamlined by working with batches of wings for faster analyses and identification results.

Moreover, this study also introduced a more practical method of GM analysis by utilizing a lesser number of landmarks on the center of the wing to describe wing shape. Nearly 300 specimens are used, fulfilling the criterion from Bookstein (30), where there are at least twice the number of individuals than landmarks. In addition, using just seven landmarks not only reduces the landmark annotation time but also addresses the limitations of GM, specifically for specimens of the order Lepidoptera. The wing margins are prone to damage due to trapping methods, such as recovery from sticky Delta traps or from traps that are not checked daily (12, 39, 40). Additionally, wing GM in Lepidoptera requires the removal of scales, and the manual cleaning process can further damage the delicate wing margins. These limitations suggest that not every collected moth can be identified using GM. However, the landmarks chosen for this study originate around the intact center of the wing. The use of the seven landmarks increases the overall applicability of the GM process. Furthermore, in this study, most of the specimens were males collected from pheromone trapping, but the analysis also included 56 female *C. includens.* The analysis of sexual dimorphism in *C. includens* specimens found no differences in wing shape, allowing the GM technique here to be applied to the identification of both female and male specimens.

Implementing GM as an identification tool requires a database of specimens that account for the intraspecific variability in wing shape. The present study used a large number of *C. includens* specimens trapped year-round in the Florida Panhandle. Previously, a study using year-round sex pheromone trapping and genitalia dissection indicated the presence of *C. includens* throughout the year, alongside a high diversity of plusiines that are cross-attracted to the commercial formulation of the *C. includens* sex pheromone lure (12). The subtropical climate of Florida, combined with the presence of host plants during the crop season and alternative hosts during the fallow season, promotes the persistence of several Neotropical plusiine species in the Panhandle agricultural landscape. The specimens used in the study achieved a sufficient representation of the species for the southeastern U.S. due to the site’s geographic positioning relative to *C. includens* phenology. In the case of *C. chalcites*, an invasive species with an expected low number of specimens available for studies, we were only able to receive and analyze 23 specimens from the APHIS survey program, which is still a sufficient sample size for GM analyses as described by Bookstein (30). The recovery of these specimens was limited to surveys in Florida and Indiana, but the origin of these insects serves as a randomized selection of the wing variability of *C. chalcites*. We believe the data used here are representative of both species for wide-scale applications of GM. However, future studies should use a larger database of native, cross-attracted plusiine species, particularly *T. ni* and *Ct. oxygramma*. Examining specimens across geographic distributions and from various host plants can improve the dataset used for GM analysis.

The validation of GM also has the potential to be used in automation for species identification in survey programs by simply requiring an image of a cleaned wing. Automating species identification of *C. chalcites* and *C. includens* can support survey programs of the invasive *C. chalcites* and serve as a novel tool for monitoring of initial infestations of *C. includens* in cultivated crops, such as soybean and peanut. Multiple studies have already applied the automation of species identification with morphometric analysis. For example, Bustamante et al. (41) developed a web-based application for streamlining the GM process of honeybee (*Apis* spp.) identification. Their program offers a user-friendly interface that simplifies GM, but users must manually annotate landmarks and align data with a Procrustes fit. In the case of *Chrysodeixis* spp. identification, users may not be precise in their landmark and Procrustes placements, which might generate incorrect results for closely related species. This user bias can be exacerbated in the GM analysis of *Chrysodeixis* spp. because of the fewer landmarks used. Another study applied machine learning tools to resolve these issues by automating the entire GM process. Their software recognizes, annotates, and analyzes the landmarks of a given honeybee image and provides classification to the subspecies level (42). Ultimately, both studies indicate that the identification of the invasive *C. chalcites* can be further optimized for survey programs and for monitoring *C. includens* in IPM programs.

In summary, the results of this study contribute to the identification of resources for pest species in the subfamily Plusiinae by providing the validated GM information to allow differentiation between the invasive *C. chalcites* and the native *C. includens*. In cases where the confirmation of detection of invasive *C. chalcites* by DNA analysis is still demanded, morphometric analysis reduces screening sets to a smaller number of samples, eliminating native species with close morphology, such as *C. includens.* It is expected that this study will be a template for continuing research, exploring other invasive species of plusiine and noctuid moths, and contributing to future automation of insect identification.

## Data Availability

The raw data supporting the conclusions of this article will be made available by the authors, without undue reservation.
